# Introducing a new proforma for the safe use of intraoperative tourniquets in orthopaedic surgery

**DOI:** 10.1308/rcsann.2023.0072

**Published:** 2024-04-05

**Authors:** M Garner, G Gaurav, Z Shahid, S Shaunak, A Vats, M Imam, T Antonios

**Affiliations:** Ashford and St. Peter’s Hospitals NHS Foundation Trust, UK

**Keywords:** Tourniquet, Guidelines

## Abstract

**Introduction:**

The routine use of pneumatic tourniquets in orthopaedic surgery is widely adopted in current practice; however, practice varies considerably based mainly on anecdotal and cultural traditions. This Quality Improvement Project evaluated current service as per the newly published British Orthopaedic Association Standards for Trauma & Orthopaedics guideline on ‘The Safe Use of Intraoperative Tourniquets’.

**Methods:**

Patient records were reviewed retrospectively for all patients who underwent orthopaedic surgery in September 2021 at one NHS hospital trust. Simultaneously, a nine-question survey was distributed to the orthopaedic teams allowing assessment of non-quantifiable aspects of the guidelines. The results were delivered as a local presentation, and trust-wide dissemination of posters using the mnemonic ‘PRESSURE’ was used to educate staff. The quantitative audit was repeated twice, after this intervention (March 2022) and after the advent of a new electronic patient record system with an online proforma (January 2023).

**Results:**

There was significant improvement (*p*<0.05) in all aspects of tourniquet documentation between the audit cycles. Maximum advised tourniquet duration was exceeded in <2% of cases regardless of guideline publication. Recommended pressures were used in less than one-third of cases in all audit cycles, with no significant change throughout. More than 50% of respondents sized their tourniquet on ‘whatever looked best fit’.

**Conclusions:**

Despite tourniquet usage being part of the UK Trauma & Orthopaedic Surgery curriculum, this study is the first to highlight a lack of compliance with ‘gold standard’ guidelines and the need for increased training for staff to ensure patients are exposed to the safest possible environment. Although electronic proformas can aid recording of information, the limitation to change is cultural tradition and anecdotal experience.

## Introduction

The routine use of pneumatic tourniquets in elective and trauma orthopaedic surgery is widely adopted in current practice. These simple, cheap devices have changed little in mechanism over the past century since Harvey Cushing introduced the pneumatic tourniquet in 1904. The device entails a cuff (sterile or non-sterile) applied over padding proximal to the surgical site, connected via tubing to a pump. The cuff is inflated to a predetermined pressure after either elevation or using an exsanguination device such as an Esmarch bandage or Rhys Davis device.

## Objective

This Quality Improvement Project (QIP) aims to evaluate current service as per the newly published British Orthopaedic Association Standards for Trauma & Orthopaedics (BOAST) guideline on ‘The Safe Use of Intraoperative Tourniquets’ and reviews whether its publication impacted practice at one busy orthopaedic department on a short-term basis and whether practice can be improved with simple interventions.^1^ This guideline is part of the British Orthopaedic Association’s (BOA) ‘elective’ guideline series, but pragmatically applies to all orthopaedic patients (elective or trauma) undergoing a procedure involving a tourniquet, excluding patients treated pre-hospitable with tourniquet application for an exsanguinating vascular injury.

## Background

Tourniquet application allows the surgeon to work within a relatively ‘bloodless’ field, improving the safety, ease of operation and precision gained by lack of visual obstruction from blood to the surgical area while limiting blood loss and the physiological sequelae faced by the patient.^2^

Tourniquets have been used throughout history, in both military and civilian populations, to control haemorrhage in life or limb-threatening injury. Arhigenes and Helidorus employed cloth strips wrapped around bleeding or amputated limbs during the Roman era.^2^ More modern devices followed, allowing higher and regulated pressure compression and controlling venous and arterial bleeding. The screw tourniquet invented by Jean Louis Petit (1674–1750) mechanically tightened the strap around the limb, creating adequate pressures to control arterial exsanguination and allowing fast release. Lister pioneered non-emergent bloodless field surgery while promoting the addition of prior limb elevation for at least 4 minutes allowing venous drainage and arteriolar constriction. Esmarch later developed his eponymous rubber bandage to exsanguinate venous blood but commented that if soft tissues contained pus, elevation alone should be used to exsanguinate the limb because of the potential risk of spreading infection.

Despite being a potentially lifesaving tool, controversies and complications have surrounded tourniquets. These include, but are not limited to, local tissue ischaemia, nerve palsies, chemical burns, pressure sores and deep vein thrombosis (DVT).^3^

Despite some guidance in the literature, there has yet to be a formal, agreed standard of care for tourniquet use among orthopaedic surgeons. Current practice varies considerably, mainly based on anecdotal and cultural traditions.^4^ In 1996, Braithwaite and Klenerman produced a modified version of Bruner's ten rules on safe tourniquet use (Table 1).^5,6^ In 2007, the North American-based Association of periOperative Nurses produced a comprehensive checklist, more specific than Bruner's original rules, aimed at theatre nurses covering preoperative assessment, tourniquet application, inflation, deflation, equipment safety and documentation.^7^ A 67-mark questionnaire based on knowledge of these principles among United Kingdom (UK) orthopaedic registrars (*n*=29) and orthopaedic department practitioners (*n*=25) demonstrated poor understanding of safe tourniquet use amongst both sets of healthcare professionals, with orthopaedic department practitioners scoring significantly higher than the surgeons.^4^ Despite being part of the UK Trauma & Orthopaedic Surgery curriculum, this study demonstrated an apparent lack of formal teaching among orthopaedic trainees, with most orthopaedic surgeons learning their tourniquet practice solely from colleagues they encounter during their training.^8^

**Table 1:** Modified version of Bruner’s ten rules on safe tourniquet use^5,6^

1	Application	Only to a healthy limb or with caution to an unhealthy limb
2	Size of tourniquet	Arm 10cm, leg >15cm
3	Site of application	Upper arm, mid/upper thigh
4	Padding	At least two layers of orthopaedic wool
5	Skin preparation	Occlude to prevent soaking of wool
6	Pressure	Arm: 50–100mmHg > SBP OR 200–250mmHg; thigh: double SBP OR 250–350mmHg
7	Time	Absolute maximum 3h, generally not to exceed 2h
8	Temperature	Avoid heating (e.g. hot lights), cool if feasible and keep tissues moist
9	Documentation	Duration and pressure at least
10	Calibration and maintenance	Weekly against mercury thermometer. Three-monthly maintenance

## BOAST recommendations

The recommendations taken from the BOAST guidelines can be divided into two subsections (Table 2):

• Documentation of use
• Safe parameters.

**Table 2:** Summary of British Orthopaedic Association Standards for Trauma & Orthopaedics guidelines^1^

Documentation	Safe parameters
Type of tourniquet	Width >50% limb diameter or contoured for patients with conical limbs Digit tourniquets should be highly visible or included in the surgical instrument count
Condition of the tourniquet site prior to and at the end of procedure	
Method of isolation used to exclude skin preparation fluids from seeping under the tourniquet	
Method of exsanguination	Compressive exsanguination contraindication in infection, history of malignancy or risk of deep vein thrombosis
Pressure	<16 years: SBP + 50–100mmHg. Lower limb adults: SBP + 70–130mmHg. Upper limb adult: SBP + 50–100mmHg.
Duration	Ideally <120min. Audible reminders every 10mins beyond 120mins
Complications	Discussion with a plastic surgical and/or tissue viability team

### Documentation of use

The Royal College of Surgeons of England’s ‘Good Surgical Practice’ stipulates surgeons should record accurate and comprehensive operation notes, allowing clear communication of information with other healthcare professionals and acting as a medicolegal document.^9^ Although not explicitly mentioned, tourniquet use is essential to the ‘operative procedure’ and should be recorded accordingly. The ‘Documentation’ column of Table 2 highlights all details that should be documented in the operative note as per BOAST, highlighting the requirement for more detailed information than in the modified Bruner's rules.

### Safe parameters

#### Duration

Tourniquets should be left inflated for the shortest period possible. The maximum time a limb can remain ischaemic before detrimental sequelae, such as muscle and nervous tissue injury, occur depends on patient factors such as comorbidities, age and baseline vascular integrity. The literature recommends a maximum of 90–120mins in healthy adults derived from when muscle adenosine triphosphate stores deplete.^10^ This period will differ between frail osteoporotic and fit paediatric patients.

#### Pressure

Tourniquet pressure balances the risks between nerve and muscle injury because of compressive forces versus failing to achieve a bloodless field. The consensus in the literature is that 300mmHg and 250mmHg for lower and upper limbs, respectively, are generally ‘safe’ pressures to achieve this. Recently, this has been criticised because it fails to account for patients' varying blood pressures, usually leading to higher than required pressures being applied, potentially increasing the risk of complications. Determining limb occlusion pressure (LOP) is considered a safer method, with systolic blood pressure (SBP) used as a surrogate marker. LOP is the minimum pressure needed to stop arterial blood flow in a given patient at a given time with a specific tourniquet. It is calculated before surgery by assessing when a Doppler signal disappears from the distal extremity as the tourniquet is inflated. A pressure of 50–100mmHg is then added to this value to use intraoperatively. In practice, this is rarely done, probably because of time constraints.^10,11^

#### Exsanguination

Varying methods exist to exsanguinate the limb before inflation. One study using labelled erythrocytes demonstrated that elevation alone is less effective than compressive methods (e.g. Esmarch, squeeze technique, roll cuff) in terms of reduction of blood volumes in the distal limb. Also, no significant increase in volume exsanguinated was seen beyond 5s of elevation.^12^ Despite no high-level evidence, because of the mechanical forces applied by exsanguinating devices, the consensus is that they should be avoided if there is a risk of disseminating tumours, infection or dislodging clots.^13^

#### Tourniquet size

The size and shape of limbs are highly variable, and there is no ‘one size fits all’. In an animal study, Ochoa demonstrated that nerve damage occurred directly beneath the cuff and suggested the damage was caused by the pressure gradient at the edge of the cuff.^14^ This implied that a narrower cuff, with less tissue directly beneath, might result in less risk of nerve damage. Conversely, other studies have suggested that a wider cuff is safer because it has an inverse relationship with LOP, hence lower pressures can be utilised.^15^

## Methods

To improve our practice, the authors carried out a QIP in their trauma unit looking at the quality of documentation and the use of tourniquets, using the criteria recommended by the BOA. The QIP was registered with the trust audit department.

The initial audit was performed retrospectively. Electronic patient records (EPR) and paper records were reviewed for all patients who underwent trauma or elective orthopaedic surgery in September 2021 across both sites at one NHS hospital trust. Simultaneously, an anonymous, voluntary, nine-question survey was distributed to all members of the orthopaedic surgical team (Foundation Year 1 doctor and above) and the orthopaedic theatre team (scrub nurses, operating department practitioners). It consisted of questions with multiple choice responses and white box answers allowing assessment of some aspects of the guidelines not amenable to quantifiable data collection methods.

**Figure 1:**
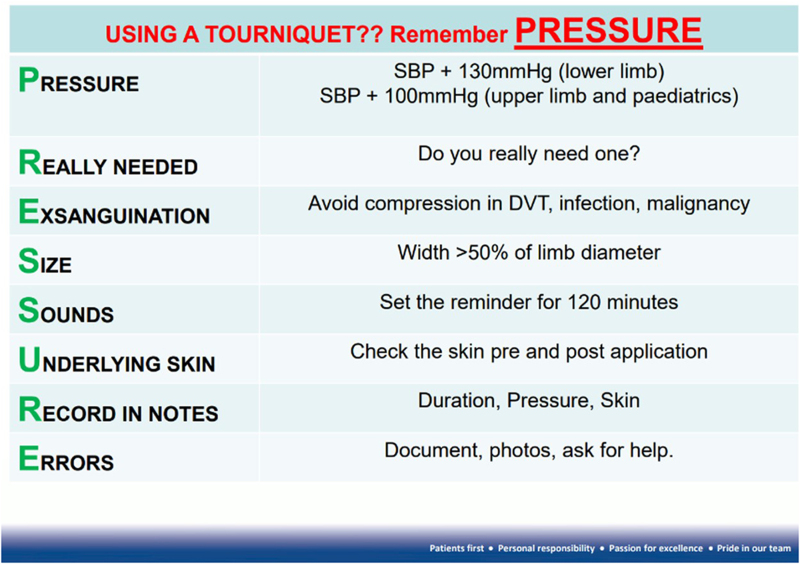
Aide memoir poster displayed in orthopaedic theatres and offices. DVT = deep vein thrombosis; SBP = systolic blood pressure

Before formally introducing the new BOAST guidelines to the trust, these two methods gave a quantitative and qualitative overview of tourniquet use in the orthopaedic department. The results were delivered as an oral presentation locally at the trust-wide Quality & Safety Half Day in December 2021. This presentation and the subsequent trust-wide dissemination of posters using the mnemonic PRESSURE (Figure 1) in operating theatres and surgeons' offices were used to highlight the publication of and educate relevant healthcare workers on the new BOAST guidelines.

The quantitative audit was repeated in the same retrospective manner, reviewing the data from EPRs for all patients who underwent orthopaedic surgery in March 2022, 6 months after the introduction of the guidelines and 3 months after the education session and poster dissemination (to avoid significant changes in rotational doctors).

**Figure 2:**
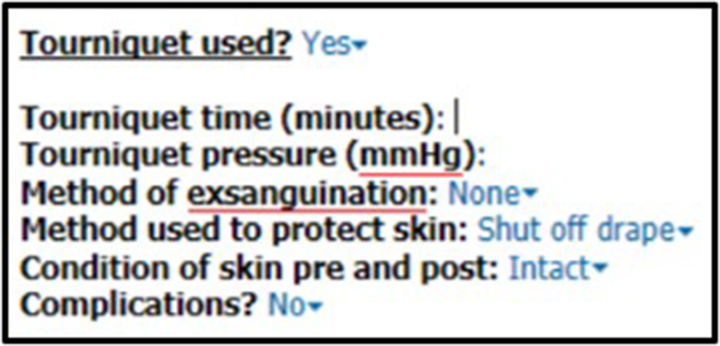
Auto-text proforma surgeons can add to their electronic operative note

In May 2022, this trust introduced a new EPR system with the abolition of handwritten operative notes. As a function of the EPR, operative note templates now have an embedded ‘tourniquet’ section that pre-populates with information inputted by the scrub team regarding tourniquet use. The authors created an additional auto-text proforma with drop-down options in line with BOAST documentation standards (Figure 2). The authors anticipated this might improve tourniquet use documentation (alternative hypothesis, H_1_). The null hypothesis (H_0_) was: ‘There will be no difference in BOAST documentation standards between the first and the third audit cycle’*.* After the first month of introduction (January 2023), a second reaudit was completed (third audit cycle) using identical methods.

**Figure 3:**
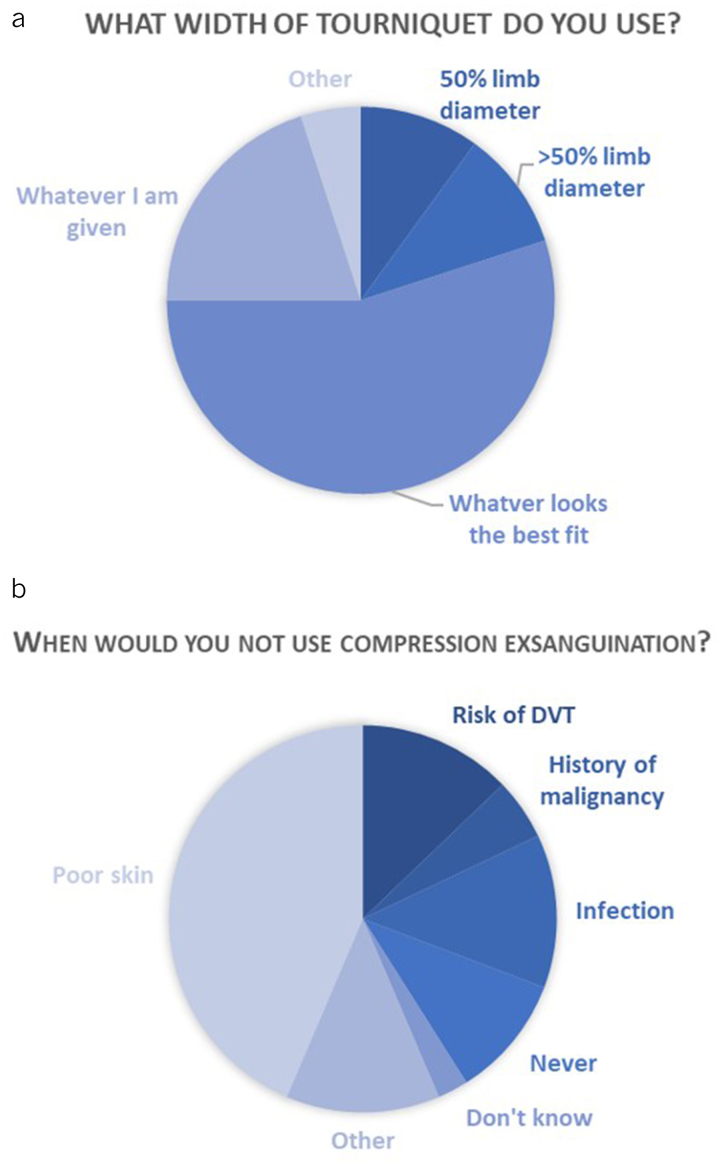
Charts illustrating results from staff survey question: (a) what width of tourniquet do you use? (*n*=19); (b) when would you not use compressive exsanguination? DVT = deep vein thrombosis, (*n*=43, respondents could state more than one response).

### Statistical analysis

The results were statistically analysed using chi-squared testing (two-tailed *p*-values calculated). ‘Documented’ vs ‘Not documented’ data served as categorical variables for statistical analysis of differences between the first and third audit cycles.

## Results

In total, 192 orthopaedic cases were performed in September 2021; 8% had notes missing from the EPR, so were excluded. Of the remaining 177, 70% utilised a limb tourniquet (*n*=124); 70 (56.5%), 46 (37.1%), 6 (4.8%) and 2 (1.6%) were lower, upper limb, paediatric and digital procedures, respectively. Regarding documentation, 5.6% of cases included method of exsanguination, 2.4% included method of isolation and 0% documented the condition of the skin pre and post-procedure. Fewer than 1% of patients had no documented tourniquet pressure, and 1.6% had no recorded tourniquet duration in any perioperative documentation.

Interestingly, the duration value was mainly only written in the scrub team notes; 23% of surgeons' notes did not mention it. The advised maximum pressure duration of 120mins was exceeded in two cases (1.6%), with no documented justification. Tourniquet pressures met the recommended limits in 24.3% of lower limb cases, 34.8% of upper limb cases and 66.7% of paediatric cases. This was calculated using the first recorded SBP on the anaesthetic chart or the admission SBP in local anaesthetic cases.

Analysis of self-reported behaviours was based on nineteen completed surveys. Most respondents worked as either an orthopaedic consultant (36.84%) or registrar (26.32%), with all respondents regularly involved in tourniquet use. There was no consensus on what width of a tourniquet to employ, with more than 50% of respondents judging size on ‘whatever looks the best fit’ (Figure 3a). Set pressures of 300mmHg for lower limb and 250mmHg for upper limb cases were most frequently used, with other pressures based on SBP quoted by a few respondents. Some 31.6% of respondents admitted not knowing what pressure should be used in paediatric patients. Two respondents stated they would avoid compressive exsanguination in patients with a history of malignancy, with five respondents feeling the same if there was a risk of DVT or infection. Most respondents stated that poor or compromised skin would be a contraindication for compressive exsanguination with other cited exceptions being in patients with sickle cell disease, patient choice and lymphoedema (Figure 3b).

More than 70% of respondents would document the pressure used, over 90% would record duration of inflation, but only 25% would record a justification for prolonged inflation duration. Although all respondents had a method to avoid retainment of digital tourniquets, once again, there was no consensus, with answers including ‘not removing the tab’, ‘bright colour tourniquet’, ‘included in surgical counts’, and ‘written on the board’.

In March 2022, 6 months after guideline publication, 159 cases utilised a limb tourniquet; 3.8% had notes missing and were excluded (*n*=153). Ninety-one (59.4%) cases were lower limb, 53 (34.6%) were upper limb, 3 (2.0%) were paediatrics and 6 were digital (3.9%). In terms of documentation, 9.8% of cases included method of exsanguination, 7.2% included method of isolation and 5.2% documented the condition of the skin pre and post procedure. Some 1.3% of cases had no recorded tourniquet duration in any perioperative documentation, with 23.5% of the surgical notes not mentioning it. In total, 1.4% of cases did not have a documented tourniquet pressure. The advised maximum pressure duration was exceeded in eight cases (5.2%) without a written justification. Tourniquet pressures met the recommended limits in 13.2% of lower limb cases, 34.0% of upper limb cases and 66.7% of paediatric cases.

In May 2022, after introduction of the electronic proforma, 267 orthopaedic cases were performed with no notes missing, 99 of which utilised a tourniquet (37%). Forty-six (47.0%), 42 (42.9%), 7 (7.1%) and 3 (3.1%) were lower limb, upper limb, paediatric and digital procedures respectively (Table 3).

**Table 3:** Summary of results between the initial audit cycle and subsequent first and second reaudits

	September 2021		March 2022		January 2023		Statistical analysis between first and third audit
Standard	No. of cases (*n*=124)	% of cases	No. of cases (*n*=153)	% of cases	No. of cases (*n*=98)	% of cases	Chi-squared results with one degree of freedom. Two-tailed *p*-value
Method of exsanguination used documented	7	5.6	15	9.8	42	42.9	χ^2^=34.341 ***p*=0.001**
Skin condition documented pre and post operation	0	0	8	5.2	63	64.3	χ^2^=79.714 ***p*=0.0001**
Shut-off drape documented	3	2.4	11	7.2	42	42.9	χ^2^=44.158 ***p*=0.0001**
**Length of tourniquet time documented**							
>120min	2	1.6	8	5.2	0	0	χ^2^=1.581 *p*=0.2087
Not in operative note	28	23.0	36	23.5	10	10.2	χ^2^=4.899 ***p*=0.0269**
Not recorded at all	2	1.6	2	1.3	0	0	χ^2^=1.581 *p*=0.2087
**Tourniquet pressure used within standard**							
Lower limb (SBP+130)	17 (*n*=70)	24.3	12 (*n*=91)	13.2	6 (*n*=46)	13.0	χ^2^=1.769 *p*=0.1835
Upper limb (SBP+100)	16 (*n*=46)	34.8	18 (*n*=53)	34.0	15 (*n*=42)	35.7	χ^2^=0.005 *p*=0.9414
Paediatrics	4 (*n*=6)	66.7	2 (*n*=3)	66.7	1 (*n*=7)	14.2	χ^2^=2.305 *p*=0.1290
Not documented	1 (*n*=122, 2 excluded as digital)	<1	2 (*n*=147, 6 excluded as digital)	1.4	3 (*n*=95, 3 excluded as digital)	3.1	χ^2^=1.584 *p*=0.2082

SBP = Systolic blood pressure

Some 42.9% of cases had the exsanguination method documented; this demonstrated a significant difference in documentation compared with the first audit (*p*=0.001). A significant difference was also seen with documentation of isolation method (42.9% of cases, *p*=0.0001) and documentation of skin condition (64.3% of cases, *p*=0.0001). All patients had a recorded tourniquet duration, with only 10.2% of cases not having this time documented in the operative note itself, a significant increase in compliance from the first audit (*p*=0.0269). No cases exceeded 120mins and once again, no complications were recorded. There was a slight deterioration in compliance of tourniquet pressure documentation; however, this was not significant (*p*=0.2082).

Tourniquet pressures met the recommended limits in 13.0% of lower limb cases, 35.7% of upper limb cases and 14.2% of paediatric cases. Compliance with BOAST guidelines was similar to the first audit.

No tourniquet-related complications were reported in any case in any audit cycle.

## Discussion

To the best of our knowledge, since the BOA published their guidelines on ‘The Safe Use of Intraoperative Tourniquets’, this is the first QIP to evaluate the level of compliance with it in an orthopaedic department and whether this can be improved with simple interventions.

Before publication, the data demonstrated reasonable documentation of tourniquet duration and pressure in the perioperative notes and, to a lesser extent, in the operative notes, but with little to no documentation of other stipulated variables, e.g. skin condition. Set tourniquet pressures made up the mainstay of practice, resulting in the majority of cases not meeting BOAST standards.

The introduction of electronic proformas significantly improved documentation quality (the null hypothesis was rejected). This is probably because of the visual prompt on the operative notes and the speed at which it could be completed compared with handwritten notes. However, alongside aide memoir posters and education, these interventions cumulatively made no significant difference to compliance with recommended tourniquet pressures.

This was a single-centre study, therefore the scope for conclusions was fairly limited and findings cannot be extrapolated across other hospital trusts. Nevertheless, with the UK government successfully reaching their target of ensuring that 90% of all National Health Service trusts had introduced an EPR by the end of 2023, the effectiveness of electronic proformas in improving documentation, demonstrated by this study, is likely to be universally beneficial.^16^

Interestingly, there was a significant difference (*p*=0.0001) in the percentage of cases utilising a tourniquet between the initial (70% of all cases) and final audit (37% of all cases). This could be for several reasons and is beyond the scope of this article. However, procedures done using a ‘wide awake, local anaesthetic, no tourniquet’ (WALANT) technique are gaining popularity, and there is an increasing body of literature meriting the advantages of total knee replacement without a tourniquet.^17,18^ “Tourniquets should only be used when clinically justified” is the leading statement in the BOAST guidelines. Whether surgeons start reducing the number or type of operations they use a tourniquet is yet to be seen.

## Conclusions

Overall, this study highlights the requirement for early education and training for surgeons and theatre staff on the safe use of tourniquets to ensure patients are exposed to the safest environment. Although electronic proformas can aid in recording information, the limitation to change in practice is the cultural tradition of pressures used, possibly based upon the 1996 modified Bruner's rules and surgeons' anecdotal experience of such pressures being safe and not leading to overt adverse outcomes while maintaining a bloodless field for them to operate. The guidelines recommend inflating to a pressure based upon SBP, but the SBP is not a fixed value. There is no recommendation on which time point in the perioperative period this should be based upon. The prospect of adjusting the tourniquet pressure throughout surgery based on a fluctuating intraoperative value seems complex and unpredictable, although future guidance or intervention may address this.

**This study has three take-home messages:**

• Limb tourniquets are helpful and vital adjuncts in orthopaedic surgery and, if used safely, can be utilised with minimal risk of complications.
• Although part of the UK Trauma & Orthopaedic Surgery curriculum, the tourniquet knowledge base among orthopaedic surgeons is mainly based on anecdotal or cultural practices rather than recognised guidelines.
• The new BOA guidelines provide a standardised, straightforward method of minimising risk when using a tourniquet, but there may be some delay in universal adoption of them among orthopaedic surgeons.
